# The role of Glial cell derived neurotrophic factor in head and neck cancer

**DOI:** 10.1371/journal.pone.0229311

**Published:** 2020-02-21

**Authors:** Hongbin Cao, Qian He, Rie von Eyben, Joshua Bloomstein, Dhanya K. Nambiar, Vignesh Viswanathan, Sonya Aggarwal, Shirley Kwok, Rachel Liang, Amanda Jeanette Koong, James S. Lewis, Christina Kong, Nan Xiao, Quynh-Thu Le

**Affiliations:** 1 Department of Radiation Oncology, Stanford University School of Medicine, Stanford, California, United States of America; 2 Department of Pathology, Stanford University School of Medicine, Stanford, California, United States of America; 3 Department of Pathology, Vanderbilt University Medical Center, Nashville, Tennessee, United States of America; 4 Department of Biomedical Sciences, University of the Pacific Arthur A. Dugoni School of Dentistry, San Francisco, California, United States of America; University of Wisconsin, UNITED STATES

## Abstract

Glial cell-derived neurotrophic factor (GDNF) is reported to promote the survival of neurons and salivary gland regeneration after radiation damage. This study investigated the effect of GDNF on cell migration, growth, and response to radiation in preclinical models of head and neck squamous cell carcinoma (HNSCC) and correlated GDNF expression to treatment outcomes in HNSCC patients. Our ultimate goal is to determine whether systemic administration of GDNF at high dose is safe for the management of hyposalivation or xerostomia in HNSCC patients. Three HPV-positive and three HPV-negative cell lines were examined for cell migration, growth, and clonogenic survival *in vitro* and tumor growth assay *in vivo*. Immunohistochemical staining of GDNF, its receptors GFRα1 and its co-receptor RET was performed on two independent HNSCC tissue microarrays (TMA) and correlated to treatment outcomes. Results showed that GDNF only enhanced cell migration in two HPV-positive cells at supra-physiologic doses, but not in HPV-negative cells. GDNF did not increase cell survival in the tested cell lines post-irradiation. Likewise, GDNF treatment affected neither tumor growth *in vitro* nor response to radiation in xenografts in two HPV-positive and two HPV-negative HNSCC models. High stromal expression of GDNF protein was associated with worse overall survival in HPV-negative HNSCC on multivariate analysis in a combined cohort of patients from Stanford University (n = 82) and Washington University (n = 189); however, the association between GDNF gene expression and worse survival was not confirmed in a separate group of HPV-negative HNSCC patients identified from the Cancer Genome Atlas (TCGA) database. Based on these data, we do not believe that GNDF is a safe systemic treatment to prevent or treat xerostomia in HNSCC and a local delivery approach such as intraglandular injection needs to be explored.

## Introduction

Head and neck cancer is the 9^th^ most common cancer globally [1, 2] and head and neck squamous cell carcinoma (HNSCC) accounts for most of these cases [3]. HNSCC could be related to alcohol and tobacco use and human papillomavirus (HPV) infection, with distinct prognosis [4–7]. Regardless of HNSCC type, most of these patients receive radiotherapy (RT) as part of their treatment, either in the definitive or adjuvant setting. Despite the widespread application of intensity-modulated RT (IMRT), which is aimed to spare the parotid glands from RT-related toxicity, the submandibular glands (SMG) are still damaged due to their close proximity to the draining cervical lymph nodes. Consequently, a large percentage of HNSCC patients still suffer from xerostomia (severe dry mouth) and its related ramifications [8].

The Glial Cell-Derived Neurotrophic Factor (GDNF) is a member of the GDNF family of ligands (GFL), which also includes Neurturin (NRTN), Artemin (ARTN), and Persephin (PSPN) [9, 10]. GDNF functions through binding to the GDNF Family Receptor-α1 (GFRα1) on the cell membrane, which further activates RET, a receptor tyrosine kinase. Alternatively, GDNF binds the Neural Cell Adhesion Molecule (NCAM) on the plasma membrane and does not require RET receptor for downstream signaling [11].

GDNF has been shown to have pronounced effects on the survival, growth, differentiation, and migration of various neuronal subpopulations [9, 10], and its role in the treatment of human Parkinson’s disease is being evaluated through clinical trials [12, 13]. We previously found that GDNF signaling played an important function in the survival of salivary stem cell after RT and its administration improved saliva production after RT in a murine model [14]. These results suggest that GDNF offers promise for the prevention or treatment of RT-induced xerostomia.

However, GDNF and its related family members have been shown to enhance tumor aggressiveness by increasing tumor cell migration [15–17] and promoting perineural invasion [18]. In laryngeal carcinoma, a type of HNSCC, higher expression of GFRα1 and ARTN was associated with more advanced pTNM stage [19]. The expression of another GFL member, PSPN, was also noted to be higher in primary oral squamous cell carcinoma compared to normal mucosa [20]. Given these adverse associations between GFL members and cancer progression, it is essential to study in detail the effect of GDNF on HNSCC before its clinical testing in treating or preventing xerostomia.

We have previously studied the effect of GDNF in one HPV-negative HNSCC cell line, SCC22A, and found that the GDNF treatment did not modify SCC22A tumor growth or response to RT [14]. Here, we report a comprehensive *in vitro* and *in vivo* investigation of the effect of GDNF on three additional HPV-negative and three HPV-positives HNSCC cell lines. We also investigate the relationship of GDNF expression and treatment outcome in three independent cohorts of HNSCC patients.

## Materials and methods

### Materials

Recombinant human GDNF was kindly provided by MedGenesis Therapeutix (Victoria, Canada).

### Cell cultures

Six HNSCC cell lines SAS, HSC4, UMSCC22B, SCC90, HMS001 and SCC47 were cultured as previously reported. Human HNSCC cell lines: SAS and HSC4 were purchased from the JCRB Cell Bank (Osaka, Japan), UMSCC1 and UMSCC22B were obtained from University of Michigan (Courtesy Dr. Carey), 93VU147T was obtained from Dr. Hans Joenje (VU Medical Center Van der Boechorststraat, The Netherlands), HMS001 was a generous gift from Dr. William A. Michaud (Mass. General Hospital Cancer Center, Boston, MA), SCC90 was a generous gift from Dr. Camille Ragin (University of Pittsburg Cancer Institute, Pittsburgh, PA), and UM-SCC47 was purchased from EMD Millipore (Temecula, CA). SAS, HSC4, UMSCC22B, SCC90, and 147T were maintained in DMEM containing 10% fetal calf serum (FCS), penicillin (100U/ml), and streptomycin (0.1mg/ml). UMSCC1 was maintained in DMEM supplemented with 10% FBS, hydrocortisone (1μg/ml), penicillin (100U/ml), and streptomycin (0.1mg/ml). HMS001 was cultured in DMEM/F12 containing 10% FBS, penicillin (100U/ml), and streptomycin (0.1mg/ml). UM-SCC47 was maintained in DMEM high Glucose, containing 10% FCS, non-essential Amino Acids, penicillin (100U/ml), and streptomycin (0.1mg/ml). All the cells were maintained in a 5% CO2 atmosphere at 37 °C.

### Antibodies

Primary antibodies against GDNF, GFRα1, RET and NCAM were purchased from Abcam (Cambridge, MA), and phospho-RET antibody was from Cell Signaling Technology (Massachusetts, USA). Secondary antibody Alexa Fluor@donkey anti-Rabbit IgG (H+L) were obtained from Life Technologies (Carlsbad, CA).

### Immunocytochemistry and image acquisition

HNSCC cells were grown on coverslips. After fixation with 4% Para-Formaldehyde/PBS, non-specific binding sites were blocked with 5% BSA in PBS for 1h at 37°C. Cells were then incubated with primary and secondary antibodies in 2.5% BSA. The coverslips were mounted with 4’, 6- diamidino-2-phenylindole (DAPI, Vector Laboratories). Immunofluorescence images were acquired using a Zeiss LSM510 laser scanning confocal microscope.

### Migration assay

*In vitro* migration assays were performed using corning transwell inserts (8- μm pore size; Corning, MA) in 24-well plates as reported [17]. Approximately 1 x 10^4^ cells were seeded in the upper chamber in serum free medium. GDNF was added in serum free medium in the lower chamber. Post-incubation, cells migrated to the underside of the filters were stained and counted under a microscope.

### Clonogenic survival assay

Clonogenic survival assay was performed as previously reported [21]. Briefly, 300 cells/plate for non-IR control, 2000 cells/plate for 4 Gy IR, and 10000 cells/plate for 8 Gy IR. Cells were plated at the number describe above and then incubated with different concentrations of GDNF or PBS 2h before RT. Colonies grew for about 7 days and then were stained with 0.5% crystal violet in methanol. Colonies with more than 50 cells were counted and survival rate was determined by the colony numbers normalized by plating efficiency. The results represent the mean of triplicate with the error bars representing ± 1 SD.

### In vitro proliferation of head and neck cancer cell lines

SCC90, HMS001 cells were plated in equal cell numbers in the presence of 100 ng/ml, 500 ng/ml GDNF or PBS and counted during logarithmic growth phase with a hemacytometer. GDNF was routinely replaced every two days.

### Animal study

All the animal studies were conducted in compliance with the Administrative Panel on Laboratory Animal Care (APLAC) guidelines and approved by the IACUC of Stanford University, under the APLAC protocol number 22680 (Research Compliance Office, Palo Alto, California, USA). Animal facility offers autoclaved food and water.

Six- to eight-week-old CB17-Prkdcscid/J mice were purchased form Jackson Laboratories. To generate subcutaneous tumors, 5 x 10^6^ (HMS001, SCC90, SAS and UMSCC22B) cells were injected into both flanks of the mice. Animals were under general anesthesia to minimize animal suffering and distress before implantation of tumor cells or radiation. We use Ketamine Hydrochloride (60mg/kg)/Xylazine (8mg/kg) and the mice were kept under warm pad until they recovered the anesthesia and were completely mobile. Tumor growth was measured every other day using a Vernier caliper until euthanized.

Mice were monitored daily. Those that appeared sick or moribund as demonstrated by increased respiratory rate, ruffle coats, inactivity for >2 days or with large tumors (> 5% body weight) before the actual endpoints or scheduled studies were euthanized using carbon dioxide. Animal home cage with mice was put into the chamber, and CO2 gas flow was regulated with a flow-meter device, so the gas displacement was about 10%-30% of the chamber volume/minute. Cervical dislocation was used as a secondary physical method to confirm death.

### Xenograft study

Xenograft study was performed as previously reported [21]. Briefly, 5x10^6^ cells were implanted into the both flanks of each SCID mouse. Mice were randomized to different treatment groups on the basis tumor size to ensure that all groups started with similar tumor sizes. Tumors between the sizes of 75 and 100 mm^3^ were irradiated. Radiation was performed using a 225 kVp cabinet x-ray irradiator filtered with 0.5 mm Cu (IC-250, Kimtron Inc.), and anesthetized animals were shielded with a 3.2-mm lead shield with a 15- to 20-mm aperture. Radiation was administered at 15 Gy. 200 μg/mouse GDNF or PBS was injected directly into the tumor 24h after RT. Tumor size was measured every 1–2 days. Tumor volume was calculated by the formula (π x length x width x height)/6.

### Patients

Criteria for patient participation included (i) newly diagnosed HNSCC, (ii) available tissue block, and (iii) willingness to sign an informed consent. All tumors were staged using the 2002 American Joint Committee on Cancer staging system.

### Ethics statement

Patient samples were collected via a protocol approved by the Stanford Institutional Review Board (IRB #17757). Patients were recruited for participation in this study when the removal of their tumor tissue was needed for either diagnostic (cancer biopsy) or therapeutic (surgical resection of their cancer) purposes. No additional invasive biopsy was required for this study.

If the patient agreed to participate in the research study, the research staff obtained written informed consent from the study participant. As much time as needed was spent with each patient to ensure they understood the study and the procedures. The potential study subjects were informed that their decision had no impact on their care at Stanford University and that they were free to withdrawal consent at any time. Potential subjects were asked to repeat back to the research coordinator their understanding of the study and procedures for participating the study. No minors were recruited for this research study.

### Tissue microarray staining and scoring

The Stanford University tissue microarray (TMA) was constructed, from formalin-fixed paraffin-embedded samples of HNSCC as previously described [22]. Immunoperoxidase stains for GDNF (citrate, 1:100; Abcam), GFRα1 (citrate, 1:100; Abcam) and RET (citrate, 1:25; Abcam) were conducted on 4 mm-thick sections of the TMA. The TMAs were and scored by a pathologist who was blinded to the clinical data; the staining was interpreted as negative (blush cytoplasmic or no tumor staining), focally positive (<70%), or extensively positive (>70%). For outcome analysis, the focally and extensively positive groups were combined together into a single positive staining group. HPV status was assessed by both in-situ hybridization (Ventana Benchmark LT automated immunostainer) and p16 immunohistochemistry staining [23]. p16 staining was scored as positive when there was staining in at least 70% of tumor cells. Since the concordance rate was high (kappa = 0.64 for the SU) between p16 and HPV assessment in patients with oropharyngeal SCC and since there were more patients with evaluable p16 tumor staining, we used p16 as a surrogate marker for HPV status in OPSCC. p16-negative OPSCC and non-OPSCC were considered HPV-negative tumors.

### Statistical analysis

Data are expressed as mean ± SEM. The migration assay, clonogenic survival, and xenograft tumor growth curve were compared using t-tests. Overall (OS) and progression-free survival (PFS) were summarized using Kaplan Meier curves and groups were compared using log-rank tests. Distant, nodal and local failure was analyzed using competing risk methodology with death as a competing risk. The groups were compared using Gray’s test. Time to event outcomes were also analyzed in Cox proportional hazards regression model in order to be able to adjust for multiple predictors. All tests were two-sided with an alpha level of 0.05 and all data was analyzed using SAS v 9.4 (SAS Institute Inc., Cary, NC, USA).

### The Cancer Genome Atlas (TCGA) database analysis

We assessed the association between GDNF, GFRα1, RET and NCAM and overall survival of head and neck cancer patients using the HNC cohort in TCGA (The Cancer Genome Atlas) database (TCGA-HNSCC provisional dataset, n = 377). To access and analyze the data we used the UCSC Xena browser (https://xenabrowser.net) using a 50% expression cut-off value. Survival data of the HNC subgroup (HPV positive and HPV negative) were extracted for analysis and generation of Kaplan–Meier curves for overall survival was also carried out using UCSC Xena Browser.

## Results

### GDNF and GFRα1 are expressed in HNSCC cell lines

**Fig 1:**
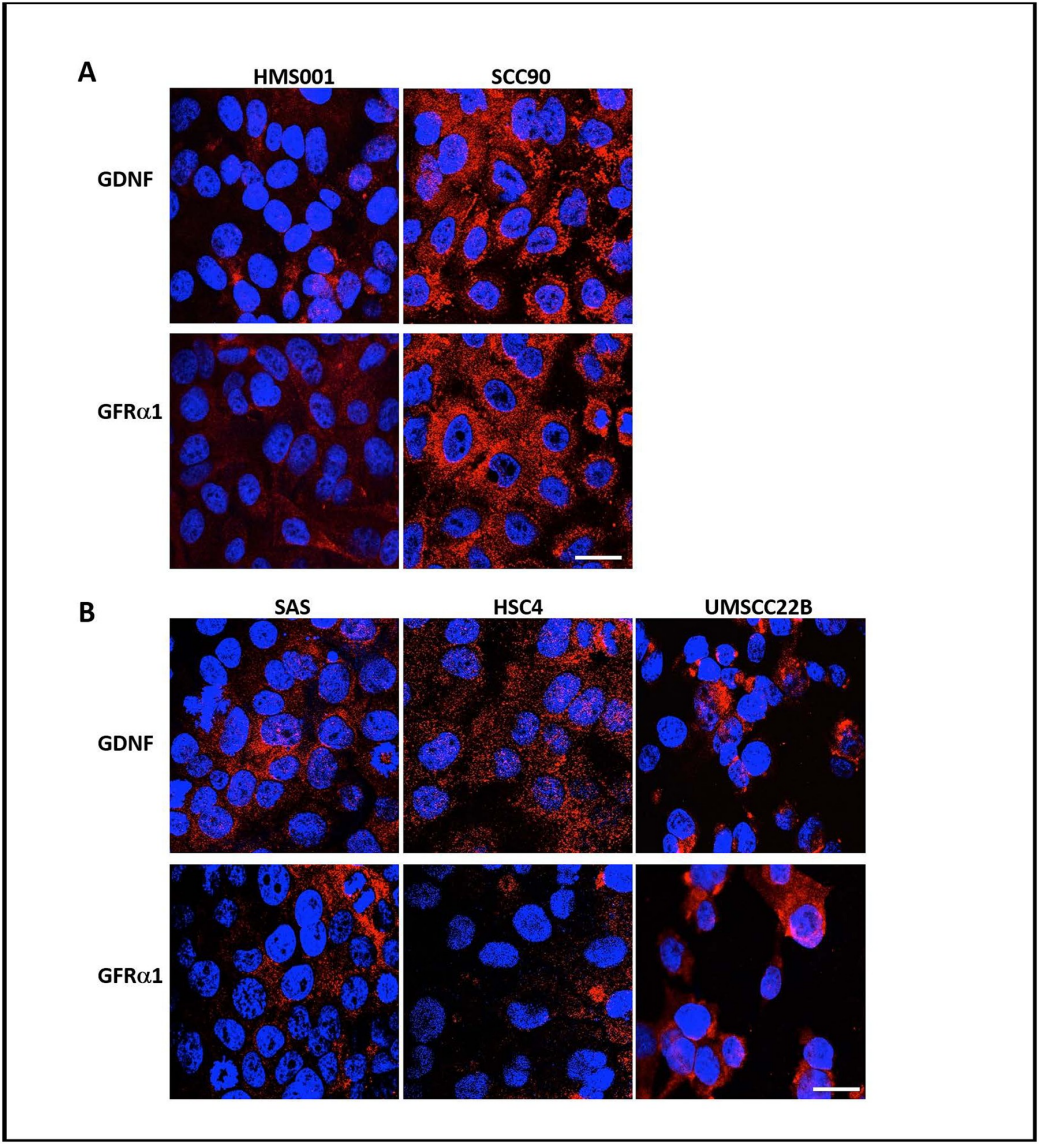
Expression levels of GDNF and GFRα1 in human HNSCC cells. Representative immunofluorescence images of GDNF and GFRα1 in HPV-positive HNSCC cell lines HMS001 and SCC90 (A) and HPV-negative HNSCC cell lines SAS, HSC4 and UMSCC22B (B). Scale bar: 20 μm.

We performed immunofluorescent (IF) staining with GDNF and GFRα1 antibodies on two HPV-positive (HMS001 and SCC90) and three HPV-negative HNSCC cell lines (SAS, HSC4 and UMSCC22B). All cell lines expressed GDNF and GFRα1 but at different levels. Between the two HPV-positive cell lines, SCC90 had higher level of both GDNF and GFRα1 compared to HMS001. Among the three HPV-negative cell lines, UMSCC22B and SAS have relatively higher GDNF and GFRα1 expression than HSC4 (Fig 1).

The expression of GDNF and GFRα1 in these cell lines was also verified by western blot analysis (S1A and S1B Fig). Of note, multiple bands are noted on the western blots for both GDNF and GFRα1 due to the existence of different isoforms, propeptides and post-translational modifications, as previously reported in the literature [24].

We also assess the expression of the GDNF’s co-receptor RET and phospho-RET (p-RET) in these cell lines. Their expressions are highly variable among the cell lines tested and do not seem to directly correlate with GDNF/GFRα1 expression (S1C Fig). Quantification of GDNF band at 24kD, GFRα1 at 51kD, p-RET and RET at 124kD is shown in S1D and S1E Fig.

We were unable to identify the expression of another GDNF’s co-receptor NCAM in the HNSCC cells using western blot. This is consistent with our previous findings that NCAM was too weak to be detected in normal un-irradiated human submandibular gland tissues, but became detectable after RT and co-localized with GDNF in the secretary duct of the salivary gland [14].

### GDNF induces migration of HPV-positive HNSCC only at high concentrations

GDNF is a potent neurotrophic factor with demonstrated ability to induce migration of non-neuronal cells, including cancer cells [15–17]. Neurotrophic factors have also been reported to promote HNSCC perineural invasion and migration [3]. To elucidate a potential link between GDNF and HNSCC migration, we performed transwell migratory assay. We selected two HPV-positive cell lines, SCC90, which has high GDNF/GFRα1 expression, and HMS001, which has low GDNF/GFRα1 expression. For the HPV-negative cell lines, we had previously studied GDNF effect on SCC22A cells (HPV-negative with high GDNF/GFRα1 expression) and reported that high dose GDNF treatment did not affect SCC22A tumor growth or response to radiation [14]. In this study, we included SAS and UMSCC22B, both of which are HPV-negative cell lines with intermediate to strong expression of GDNF/GFRα1 expression.

**Fig 2:**
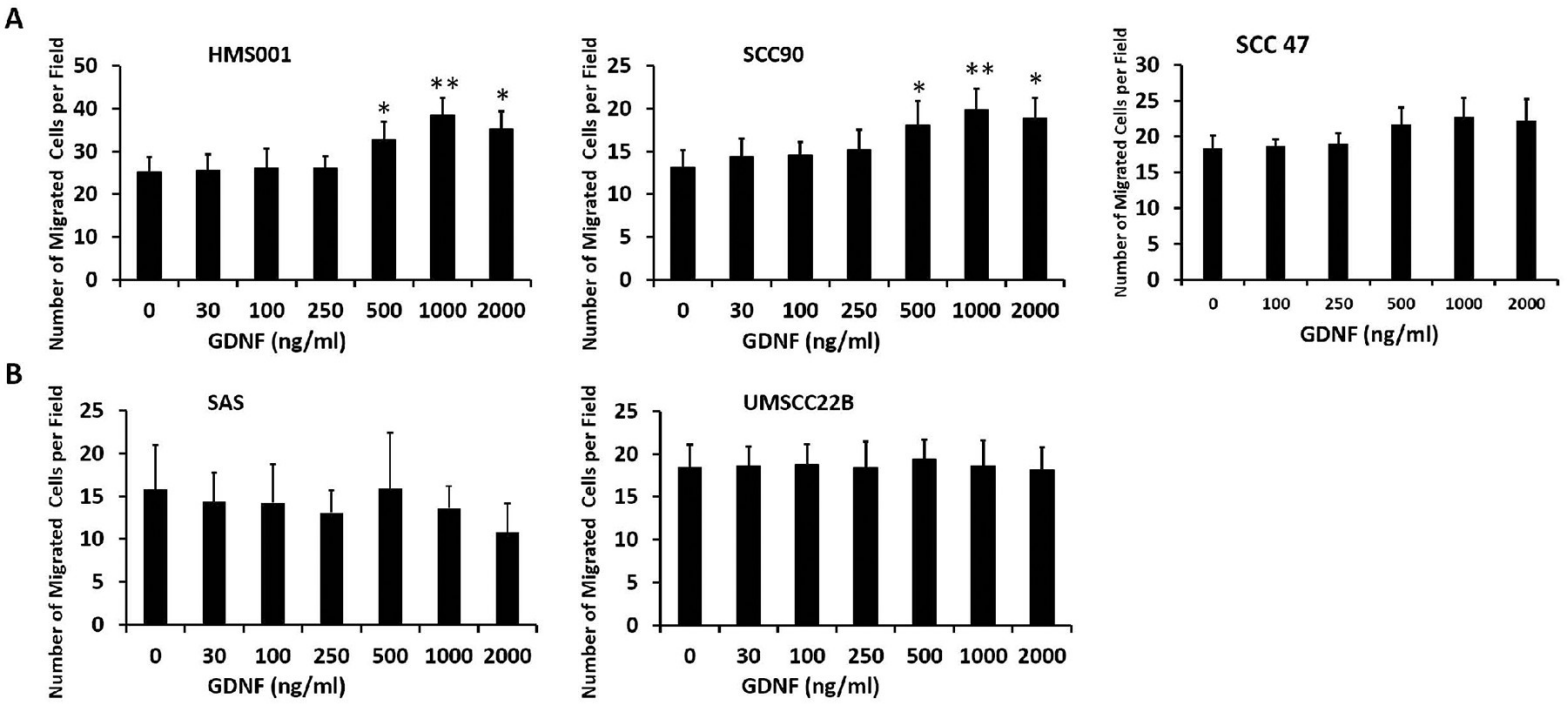
The effect of GDNF on the migratory activity of HNSCC cells. HPV-positive HNSCC HMS001, SCC90 and SCC47. (A), and HPV-negative HNSCC SAS and UMSCC22B (B) cells were incubated with various concentration of GDNF. Transwell migration was measured after 16 hrs. Results are expressed as the mean ± S.E.M. of at least three independent experiments. *p<0.05, **p<0.01.

The addition of exogenous GDNF at or below 250 ng/ml did not increase migration of SCC90 and HMS001 (Fig 2). However, at supra-physiologic doses of 500 ng/ml and 1000 ng/ml, GDNF increased migration of the two HPV-positive cell lines and the difference was statistically significant compared to vehicle control. Of interest, at 2000 ng/ml, the pro-migratory effect of GDNF plateaued or decreased, suggesting a saturation effect. We performed the same experiment on an additional HPV positive cell line SCC47. Although there was a trend for increased cell migration at GDNF doses > 250 ng/ml, the difference was not statistically significant (Fig 2).

In contrast, GDNF did not affect the migration of the HPV-negative cell lines tested at any dose ranging from 30–2000 ng/ml (Fig 2). These results are consistent with our previous finding on SCC22A cell line, which is HPV-negative.

### GDNF did not affect HNSCC tumor growth *in vitro or in vivo* after treatment with irradiation

**Fig 3:**
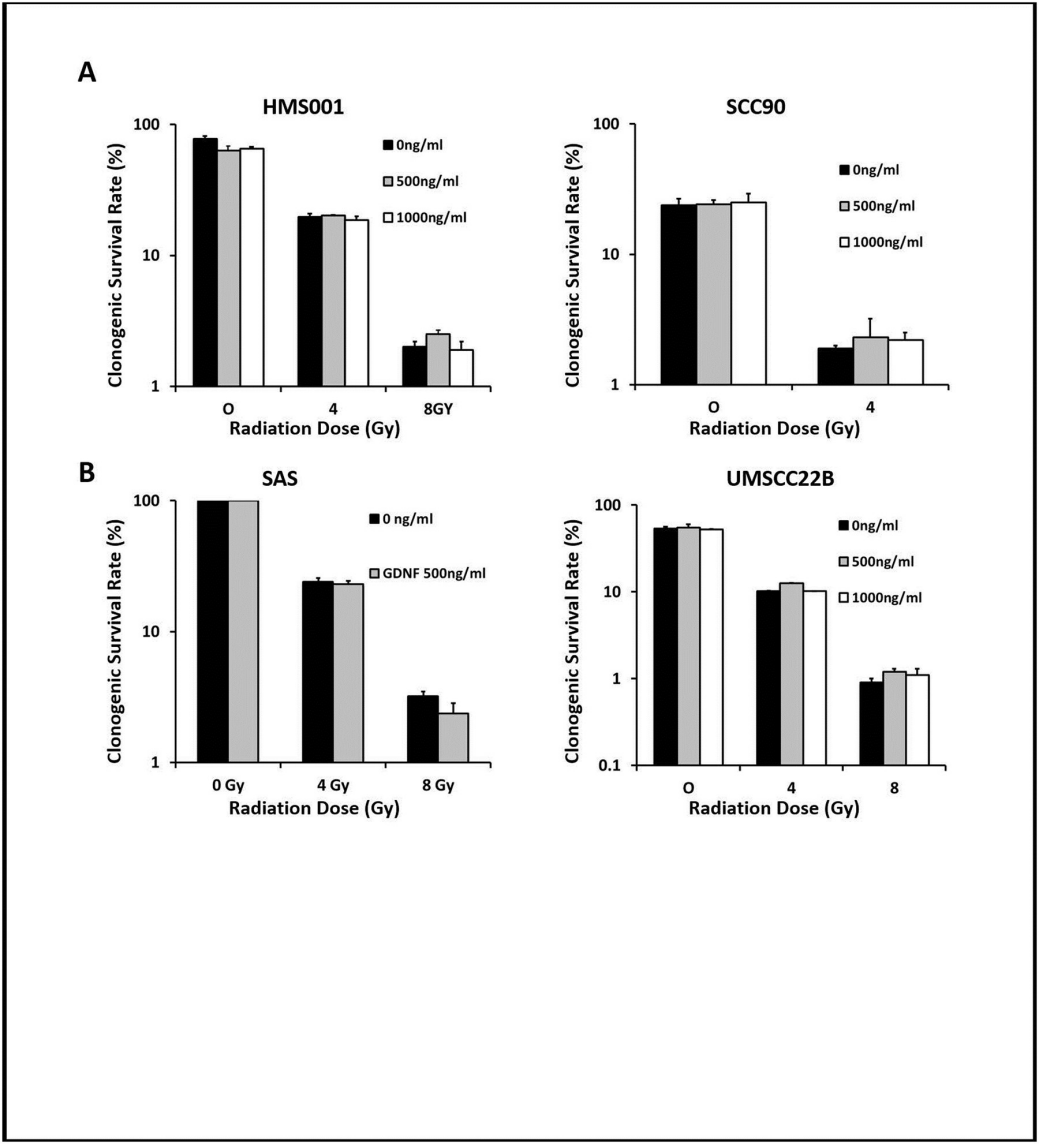
The effect of GDNF on clonogenic cell survival in irradiated human HNSCC cells. HPV-positive HMS001 and SCC90 cells, and HPV-negative SAS and UMSCC22B cells were preincubated with various concentration of GDNF (0, 500, 1000 ng/ml) for 2 hours and then irradiated with indicated radiation doses. Data represents the average of three experiments. Error bars indicate standard deviation.

To investigate the effect of GDNF on radiotherapy-induced cell killing, we conducted clonogenic survival assays on the above HNSCC cell lines. GDNF treatment did not protect HMSCC001, SCC90, SAS or UMSCC22B cells from RT, regardless of endogenous GDNF/GFRα1 expression levels or HPV status (Fig 3).

**Fig 4:**
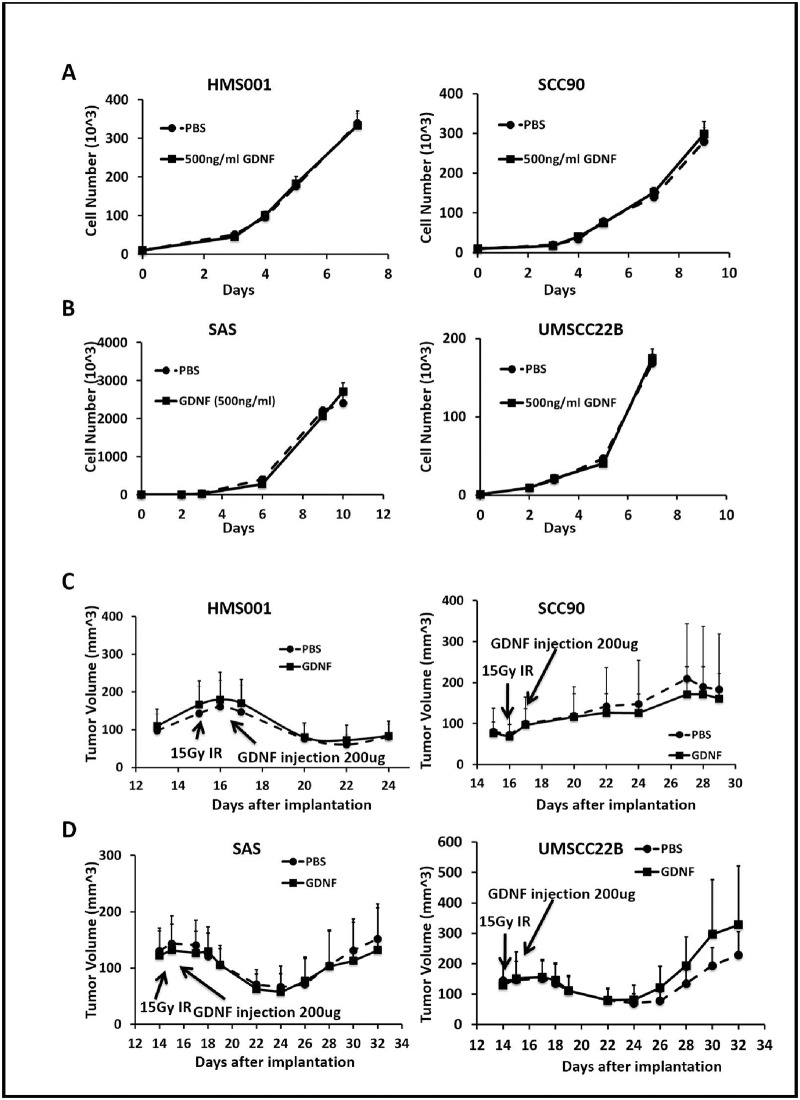
GDNF did not affect HNSCC growth *in vitro* and *in vivo*. Cell growth curves in culture for HPV-positive HMS001 and SCC90 cells (A), HPV-negative HNSCC SAS and UMSCC22B (B) treated with PBS or 500 ng/ml GDNF. (C) Volume of HPV-positive HMS001 and SCC90 tumor and (D) HPV-negative SAS and UMSCC22B tumor before and after 15 Gy irradiation and GDNF treatment. 200ug GDNF or PBS was injected 24 hours after irradiation. T-test, n = 10 tumor per group. Data was presented as mean ± S.E.M.

We also evaluated the effect of GDNF on cell proliferation *in vitro* in two HPV-positive cell lines, HMS001 and SCC90 (Fig 4A), and two HPV-negative cell lines SAS and UMSCC22B (Fig 4B). GDNF treatment did not accelerate the growth rate as compared with PBS control.

To evaluate the effect of GDNF on HNSCC growth and response to radiation *in vivo*, we performed xenograft studies with two HPV-positive cell lines, SCC90 and HMS001, and two HPV-negative cell lines, SAS and UMSCC22B in SCID mice. When the tumors reached ~100 mm^3^, we irradiated them with a single dose of 15 Gy while the rest of the body was shielded. Either 200 μg GDNF or an equal volume of PBS was injected into tumors 24 hours after irradiation. There was no significant difference between the tumor growth curves of GDNF- and PBS- treated groups for any cell line (Fig 4C and 4D). There was a slight trend for faster growth with GDNF at the later time point for UMSCC22B cells but the difference did not reach statistical significance.

### Relationship between GDNF expression and prognosis in HNSCC patients

To determine whether the expression of GDNF correlates with outcome in HNSCC patients, we first stained a tissue microarray (TMA) from Stanford University (SU) with GDNF antibody. We started out with 96 HNSCC patients from the Stanford cohort. In the p16 positive group, we excluded 2 patients with missing GDNF staining (i.e. core loss or lacking stroma in the core), and 3 patients with p16-positive non-OPSCC tumors due to unclear prognosis. In the p16 negative group, we excluded 9 patients missing GDNF staining. Therefore, 82 patients from the SU cohort (36 p16-positive and 46 p16-negative patients) were analyzable.

For the Washington University (WU) cohort, we started with 360 HNSCC patients. A total of 171 patients were excluded using the same criteria above (primarily core loss), resulting in 189 analyzable patients (122 p16-positive and 67 p16-negative patients). Of note, the WU TMAs had 47.5% missing tumor cores or uninterpretable readings as the result of sectioning for several previous studies, exhausting many tumor cores on the TMAs.

The majority of the tumors show stromal staining (S2A Fig); positive GDNF staining of tumor cells was noted in only 7 of 82 patients in the SU cohort and 45 of 189 patients in the WU cohort. Therefore, we focused the analysis on GDNF stromal staining. Patients with HPV-positive and HPV-negative tumor were assessed separately since they have distinct prognosis. We also analyzed the HNSCC patient cohort in the Cancer Genome Atlas Program (TCGA) for the prognostic significance of gene expression of GDNF and its receptors including GFRα1, NCAM and RET. Of the 604 patients in the TCGA cohort, we focused on 377 patients who have a complete set of information including age, gender, tumor site, nodal and tumor staging classification, GDNF, NCAM, GFRA and RET mRNA expression data for multivariate analysis.

S1 Table shows the patient distribution for the SU, WU and TCGA analyzable cohorts. The tumor and treatment distributions are quite different between the three cohorts and several parameters such as HPV status and treatment were missing for a large percentage of patients in the TCGA cohort. In addition, survival curves are also different between the three cohorts (S3 Fig).

We first performed univariate analysis on GDNF stromal protein expression (positive or negative) and survival. Positive stromal GDNF staining was associated with worse overall survival (OS) in patients with HPV-positive (n = 36) and HPV negative (n = 46) tumors in the SU cohort (S4A and S4B Fig). However, these observations were not confirmed in the larger WU cohort (122 HPV-positive and 67 HPV-negative tumors (S4C and S4D Fig).

**Fig 5:**
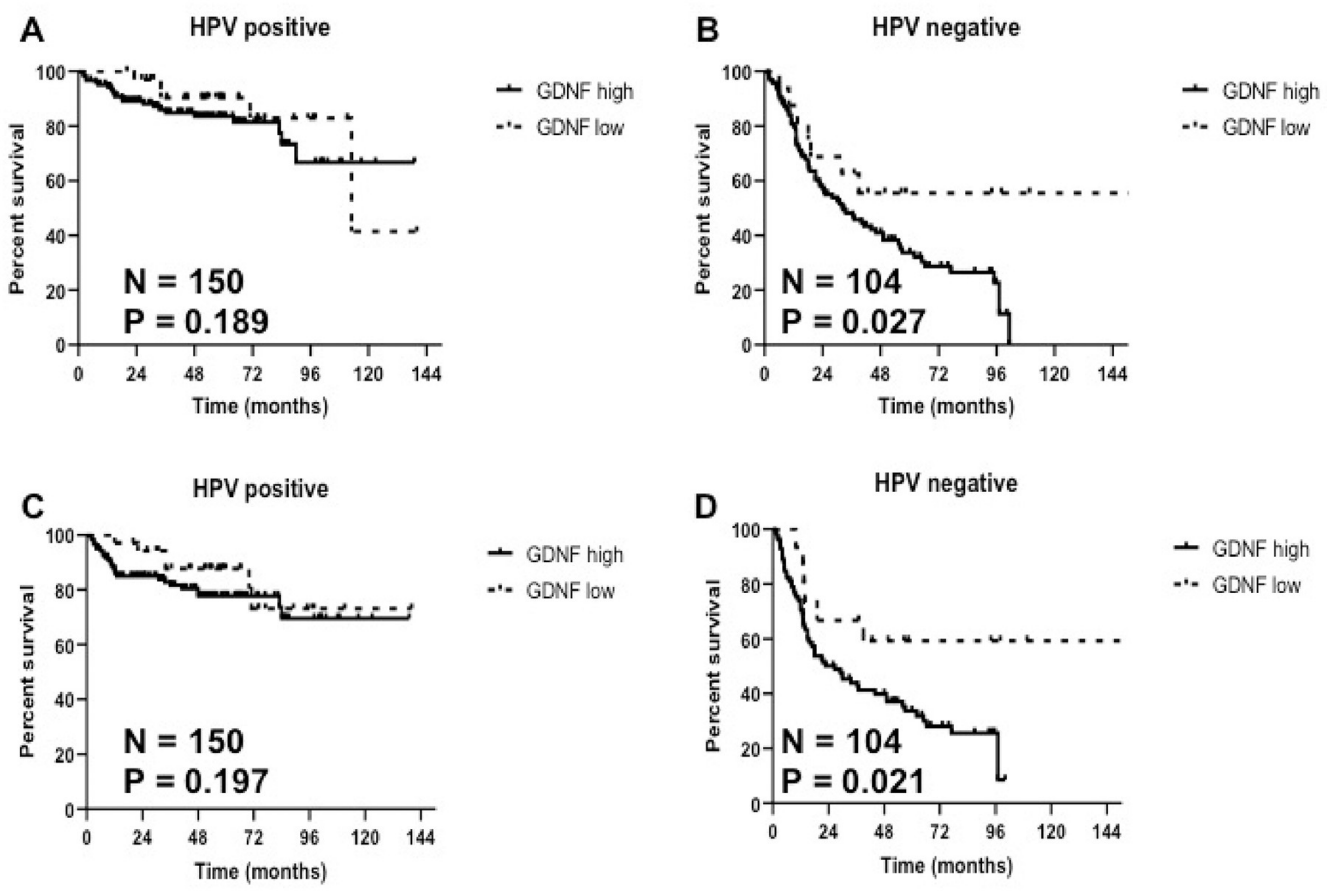
Kaplan-Meier estimates and competing risk analysis of overall survival by GDNF stromal protein expression in the combine SU/WU group for HPV-positive HNSCC **(A)**, HPV-negative HNSCC **(B)**. Kaplan Meier estimates and competing risk analysis of PFS by GDNF stromal protein expression in the combine SU/WU group for HPV-positive HNSCC **(C)**, HPV-negative HNSCC **(D)**.

Since the OS curves of the SU and WU cohort are different, likely due to differences in the stage distribution, treatment and follow-up, we combined the two cohorts (SU/WU group) to perform multivariate analyses to address for potential confounders. In addition, since HPV-positive and HPV-negative cancers are considered different entities, we performed multivariate analyses for these tumors separately. Of 150 patients with HPV-positive HNSCC in the SU/WU group, there were only 26 events, which provided enough power to fit a Cox model with three predictors. We used a stepwise selection algorithm to indicate which three predictors should be included in the model and the predictors selected were: N-stage, treatment and GDNF status. The predictor GDNF status did not reach statistical significance (hazard ratio [HR], p = 0.189, Table 1A). Of the 104 patients with HPV-negative HNSCC in the SU/WU group, there were 70 events which provided enough power to fit a Cox model with 7 predictors. We used a stepwise selection algorithm to indicate which seven predictors should be included in the model and the predictors selected were: sex, age, N-stage, treatment, T-stage, institution and GDNF status. The predictor GDNF status was significant indicating worse survival for patients with positive GDNF stromal staining (HR 2.7, p = 0.027, Table 1B). Fig 5A and 5B show OS curves for HPV-positive and -negative patients, respectively, in the SU/WU group by GDNF expression.

**Table 1:** Multivariate Cox analysis of overall survival.

A: For patients with HPV-positive HNSCC in the SU/WU group (N = 150)						
**Parameter**		**Reference**	**HR**	**95%CI**		**p-value**
**N stage**	N2	N0/N1	2.134	0.628	7.254	0.050
**N stage**	N3	N0/N1	5.812	1.336	25.276	
**Treatment**	RT+surgery/surgery only	RT only	2.954	1.292	6.754	0.010
**GDNF**	Positive	Negative	1.948	0.719	5.278	0.189
B: For patients with HPV-negative HNSCC in the SU/WU group (N = 104)						
**Parameter**		**Reference**	**HR**	**95%CI**		**p-value**
**Sex**	Male	Female	1.295	0.634	2.643	0.479
**Age**			1.014	0.990	1.039	0.260
**N stage**	N2	N0/N1	1.104	0.656	1.857	0.322
**N stage**	N3	N0/N1	1.876	0.821	4.288	
**T stage**	T3/4	T1/T2	2.114	1.190	3.755	0.011
**Treatment**	RT+surgery/surgery only	RT only	2.525	1.423	4.478	0.002
**GDNF**	Positive	Negative	**2.708**	1.119	6.556	**0.027**
**Institution**	Wash U	Stanford	1.109	0.653	1.882	0.702
C: For patients with HPV-negative HNSCC in the TCGA cohort (n = 340)						
**Parameter**		**Reference**	**HR**	**95%CI**		**p-value**
**Sex**	Female	Male	1.100	0.777	1.558	0.590
**Age**			1.023	1.007	1.039	0.005
**T stage**	T3/4	T1/2	1.724	0.979	3.037	0.059
**Stage**	Stage 3/4	1 / 2	0.751	0.386	1.461	0.398
***GDNF***	*GDNF* < 1.9	*GDNF* ≥ 1.9	**1.474**	1.063	2.044	**0.020**
**N stage**	N2/N3	NX/N0/N1	2.010	1.411	2.861	< 0.001

We next tried to correlate the gene expression of *GDNF* to overall survival in the TCGA cohort. Since HPV status is missing in 81% of the patients and our multivariate analysis for the SU/WU group suggested that GDNF protein expression may be linked to worse survival in the HPV-negative group, we focused on HPV-negative tumors in TCGA. HPV-negative tumors were defined here as those located outside of the oropharynx or those with documented HPV-negativity. 340 of 377 patients had HPV-negative tumor. The median expression was used as the cut point. Interestingly, in contrast to the IHC staining data, patients with lower *GDNF* gene expression had significantly worse survival than those with higher expression on multivariate analysis (Table 1C).

We also evaluated progression free survival (PFS), risk for local (LF), nodal (NF) and distant failure (DF) in HPV-positive and HPV-negative tumors by GDNF stromal expression in both SU (S2 Table) and WU cohort (S3 Table) where these data are available. Although positive stromal GDNF staining was significantly associated with worse PFS in the SU cohort, regardless of HPV status, this association was not observed in the WU cohort. Multivariate analysis of the combined SU/WU group showed similar results as for OS. Positive GDNF stromal protein expression was associated with significantly worse PFS compared to negative expression in patients with HPV-negative HNSCC (HR 2.8, p = 0.021, S4B Table, Fig 5D); however, the difference did not reach statistical significance in HPV-positive HNSCC (HR 1.8, p = 0.197, S4A Table, Fig 5C).

We stained the GDNF receptor GFRα1 and the co-receptor RET in the SU and WU patients (S2B and S2C Fig). GFRα1 staining was poor and not evaluable in either TMA. RET staining was poor and not evaluable in the WU cohort. In the SU cohort, positive RET staining of tumor cells was noted in only 7 HPV-positive and 1 HPV-negative patients. Therefore, we analyzed the RET stromal staining and prognosis in the SU cohort. There was no association between RET stromal staining and prognosis (S5 Table). Similarly, we did not find any correlation between overall survival and the gene expression of *GFRα1*, *NCAM or RET* in the HPV-negative HNSCC patients in the TCGA database (S6 Table).

## Discussion

Our previous study suggested that recombinant GDNF (rGDNF), when administered intraglandularly either before or after RT, was able to improve the murine submandibular gland morphology and function from RT damage, indicating that GDNF could be a potential therapeutic agent to prevent RT-related xerostomia in HNSCC patients [14]. However, because neurotrophic factors have been reported to play a potential role in cancer aggressiveness [3], we performed a systematic preclinical and clinical evaluation of the effect of GDNF in HNSCC through the use of several different cell lines and patient cohorts.

Our preclinical evaluations suggested that GDNF has minimal effect on cell proliferation or migration at the tested doses below 250 ng/ml, which is above the physiological level of GDNF in serum (711.5+/- 186.5 pg/ml) [25]. It only enhanced cell migrations in HPV-positive cell lines at supra-physiologic doses greater than 500 ng/ml. It is interesting that GDNF showed selective effect on HPV-positive cell lines HMS001 and SCC90 on promoting cell migration. However, the pattern was not obvious in another HPV–positive cell line UMSCC47. More work is warranted to further investigate the association between GDNF and HPV status of HNSCC cell lines. These data are in contrast to previously published reports where treatment with as little as 10–30 ng/ml GDNF increased migration in cancer cells [15–17]. Of note, only the study by Chuang *et*. *al*. evaluated cell migration in HNSCC cell lines HSC3 and SCC4 [17]; whereas the other two studies focused on glioma and colorectal cancer, respectively [15, 16]. The source of rGDNF is different between our study and that in Chuang’s study. We used human rGDNF protein from MedGenesis Therapeutix Inc., whereas Chuang et al used the human rGDNF from PeproTech (Rocky Hill, NJ) [17]. To address this difference, we also tested rGDNF from PeproTech; at doses < 30 ng/ml, we did not observe enhanced cell migration in the HPV-positive cell line SCC90.

The fact that GDNF treatment did not affect clonogenic survival *in vitro* or tumor regrowth after radiation *in vivo* in different tested HNSCC cell lines was promising. It is also consistent with the data that we had previously reported for SCC22A, an HPV-negative cell line. However, existing literature suggested that the GDNF family may play a role in tumor progression, prompting further evaluation of GDNF’s role in the prognosis of human HNSCC. Immunohistochemical (IHC) staining of a TMA containing 145 HNSCC patients showed that GDNF expression was significantly correlated with PD-L1 level through activation of JNK2-STAT1 signaling pathway. In addition positive GDNF expression was associated with increased perineural invasion, lymphatic metastasis and decreased overall survival of patients [26]. In laryngeal squamous cell carcinoma, the expression of GFRα1 and ARTN, another GFL member, was associated with more advanced pTNM stage [19]. GFL member PSPN has been suggested to play a key role in human oral cancer progression [20]. In human pancreatic adenocarcinoma, soluble GFRα1 was shown to be released from nerves and interacted with soluble GDNF to enhance perineural invasion, an event that is also observed in HNSCC [18]. The reports suggest that there is still a possibility for GDNF to be linked to cancer cell aggressiveness.

These data prompted us to evaluate the prognostic significance of GDNF and its receptors in human HNSCC using available TMAs from two different institutions. In the smaller Stanford cohort, regardless of the HPV status, positive GDNF stromal staining correlated with lower OS and PFS in the evaluable patients. However, this was not observed in the larger WU cohort. Because of the difference in patient distribution, follow up time and treatment approaches, we pooled the patients together to performed multivariate analysis to account for potential confounders. Interestingly, positive GDNF stromal protein expression was associated with a 2.7 folds higher risk of death compared to negative expression in the HPV-negative group. Although the difference did not reach statistical significance in the HPV-positive group, the trend was in the similar direction with a hazard ratio of ~2 favoring GDNF-negative expression. The lack of statistical significance may be due to fewer events in HPV-positive HNSCC patients.

We also analyzed a third cohort of HNSCC patients in the TCGA database, focusing primarily on those with HPV-negative HNCC, which constitute the largest group in this dataset. Interestingly, higher *GDNF* gene expression was associated with better outcome, which is opposite of the findings for protein expression. The discrepancy may be due to the fact that TCGA measures gene expression of the tumor bulk, which is comprised of cancer and non-cancer cells in the tumor microenvironment; whereas the IHC data primarily focus on GDNF stromal protein staining. In addition, protein and gene expression may vary due to translational process, protein stability, secretion and contributions from other cells within the tumor microenvironment.

Because of discrepant findings and potential drawbacks for each of these cohorts (small size for the SU cohort, high percentage of core loss for the WU cohort, high percentage patients without HPV status and/or treatment details in the TCGA cohort, retrospective nature of all cohorts), conservative interpretation of these results is therefore warranted. Base on the currently available data and previously reported links between the GFL family members and tumor aggressiveness in solid cancers, including HNSCC, and our noted negative prognosis between GNDF stromal protein expression and overall survival in HPV-negative HNSCC, we do not recommend the systemic administration of GDNF for the treatment of RT-related xerostomia. However, this does not preclude intraglandular administration of the drug as a potential route of delivery. Prior studies where GDNF was delivered through intraputamen infusion to Parkinson patients showed that that dose as high as 30 ug/day at successive 8-week intervals could be delivered without any systemic toxicity and minimal level of the protein detected in the blood [27]. Additional work will need to be conducted to determine the optimal dose and frequency of GDNF intraglandular injection to produce sustained long-term improvement of saliva production after RT damage.

Ours is the first study to evaluate the effect of GDNF on radiation response in HNSCC xenografts and one of the few to correlate GDNF expression to treatment outcome in HNSCC patients. A larger validation study will need to be performed in prospectively treated patients on clinical trials.

## Conclusion

In summary, GDNF only promotes HNSCC cell migration at supra-physiologic doses and does not protect HNSCC from RT cell kill in the preclinical setting. However, positive stromal protein expression of GDNF may be associated with a higher risk of relapse in certain patients, especially those with HPV-negative HNSCC; therefore, systemic administration of GDNF for the goal of xerostomia treatment is not recommended.

## Supporting information

S1 Fig Expression level of endogenous GDNF and its receptors in HNSCC cell lines by western blot. (A) GDNF homodimerizes and has 5 isoforms along with a signal peptide. Three major bands were observed on GDNF blot, with the 24 kDa band was the predicted molecular weight of GDNF, the band about 35 kDa could be glycosylated GDNF and the band right above 10kDa could be a signal peptide of GDNF [24]. All three bands could be blocked by GDNF peptide pre-incubation with the antibody. (B) GFRα1 has two isoforms and three glycosylation sites. We observed the apparent bands of GFRα1 with or without glycosylation (45–60 kDa) and 75–100 kDa bands. This is consistent with the previous report which indicates that the bands with high molecular mass (75–100 kDa) probably represents a mixture of GDNF monomer and dimer cross-linked to heterogeneously glycosylated GFRα1 monomer [28]. (C) GDNF’s co-receptor RET and phosphorylated–RET expression in HNSCC. (D-F) Quantification of the expression levels of GDNF, GFRα1, phosphorylated–RET and RET.
(DOCX)

S2 Fig Representative images of GDNF (A), GFRα1 (B) and RET (C) immunohistochemistry staining on HNSCC tissue microarray. GFRa1 staining showed high background and not evaluable.
(DOCX)

S3 Fig Kaplan-Meier estimates of overall survival for the SU, WU and TCGA patient cohorts in supplementary Table 1. (DOCX)

S4 Fig Kaplan-Meier estimates and competing risk analysis of clinical outcomes by HPV and GDNF stromal levels in SU and WU cohorts. Overall survival (OS) in HPV-positive tumor in SU cohort **(A)**, WU cohort **(C)**. Overall survival (OS) in HPV-negative tumor in SU cohort **(B)**, WU cohort **(D)**.
(DOCX)

S1 Table Clinical and demographic features of the Stanford University, Washington University and TCGA HNSCC patient cohorts. (DOCX)

S2 Table Statistical analysis of GDNF stromal expression in HPV positive and negative patients in Stanford University cohort. (DOCX)

S3 Table Statistical analysis of GDNF stromal expression in HPV positive and negative patients in Washington University cohort. (DOCX)

S4 Table Multivariate Cox analysis of progression-free survival. **(A)** For patients with HPV-positive HNSCC in the SU/WU group (N = 150). **(B)** For patients with HPV-negative HNSCC in the SU/WU group (N = 104).
(DOCX)

S5 Table Statistical analysis of RET stromal expression in HPV positive and negative patients in Stanford University cohort. (DOCX)

S6 Table Multivariate Cox analysis of overall survival for HPV-negative HNSCC patients in TCGA (N = 340). **(A)** Comparing *NCAM* gene expression. **(B)** Comparing GFRα1 expression, and **(C)** Comparing *RET* gene expression.
(DOCX)
