# Supplementary material for: The role of Glial cell derived neurotrophic factor in head and neck cancer
Source: PLoS One. 2020 Feb 21;15(2):e0229311. doi: 10.1371/journal.pone.0229311 (PMC7034888; doi:10.1371/journal.pone.0229311)

**Supplementary Figure 1.** Expression level of endogenous GDNF and its receptors in HNSCC cell lines by western blot. (A) GDNF homodimerizes and has 5 isoforms along with a signal peptide. Three major bands were observed on GDNF blot, with the 24 kDa band was the predicted molecular weight of GDNF, the band about 35 kDa could be glycosylated GDNF and the band right above 10kDa could be a signal peptide of GDNF [24]. All three bands could be blocked by GDNF peptide pre-incubation with the antibody. (B) GFRα1 has two isoforms and three glycosylation sites. We observed the apparent bands of GFRα1 with or without glycosylation (45-60 kDa) and 75-100 kDa bands. This is consistent with the previous report which indicates that the bands with high molecular mass (75-100 kDa) probably represents a mixture of GDNF monomer and dimer cross-linked to heterogeneously glycosylated GFRα1 monomer [27]. (C) GDNF’s co-receptor RET and phosphorylated –RET expression in HNSCC. (D-F) Quantification of the expression levels of GDNF, GFRα1, phosphorylated –RET and RET.


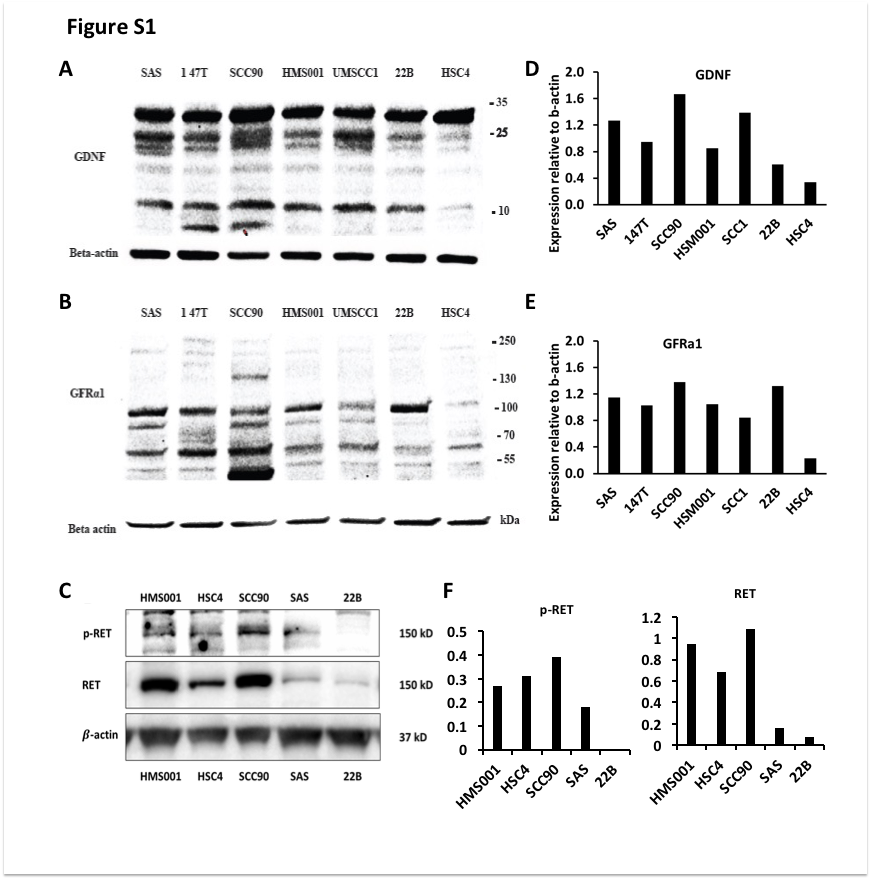

Supplement: S1 Fig — (A) GDNF homodimerizes and has 5 isoforms along with a signal peptide. Three major bands were observed on GDNF blot, with the 24 kDa band was the predicted molecular weight of GDNF, the band about 35 kDa could be glycosylated GDNF and the band right above 10kDa could be a signal peptide of GDNF [24]. All three bands could be blocked by GDNF peptide pre-incubation with the antibody. (B) GFRα1 has two isoforms and three glycosylation sites. We observed the apparent bands of GFRα1 with or without glycosylation (45–60 kDa) and 75–100 kDa bands. This is consistent with the previous report which indicates that the bands with high molecular mass (75–100 kDa) probably represents a mixture of GDNF monomer and dimer cross-linked to heterogeneously glycosylated GFRα1 monomer [28]. (C) GDNF’s co-receptor RET and phosphorylated–RET expression in HNSCC. (D-F) Quantification of the expression levels of GDNF, GFRα1, phosphorylated–RET and RET. (DOCX) [file pone.0229311.s001.docx]
