# Supplementary material for: The role of Glial cell derived neurotrophic factor in head and neck cancer
Source: PLoS One. 2020 Feb 21;15(2):e0229311. doi: 10.1371/journal.pone.0229311 (PMC7034888; doi:10.1371/journal.pone.0229311)
Supplement: S2 Fig — GFRa1 staining showed high background and not evaluable. (DOCX) [file pone.0229311.s002.docx]

**Figure S2.** Representative images of GDNF **(A)**, GFRα1 **(B)** and RET **(C)** immunohistochemistry staining on HNSCC tissue microarray. GFRa1 staining showed high background and not evaluable.

**
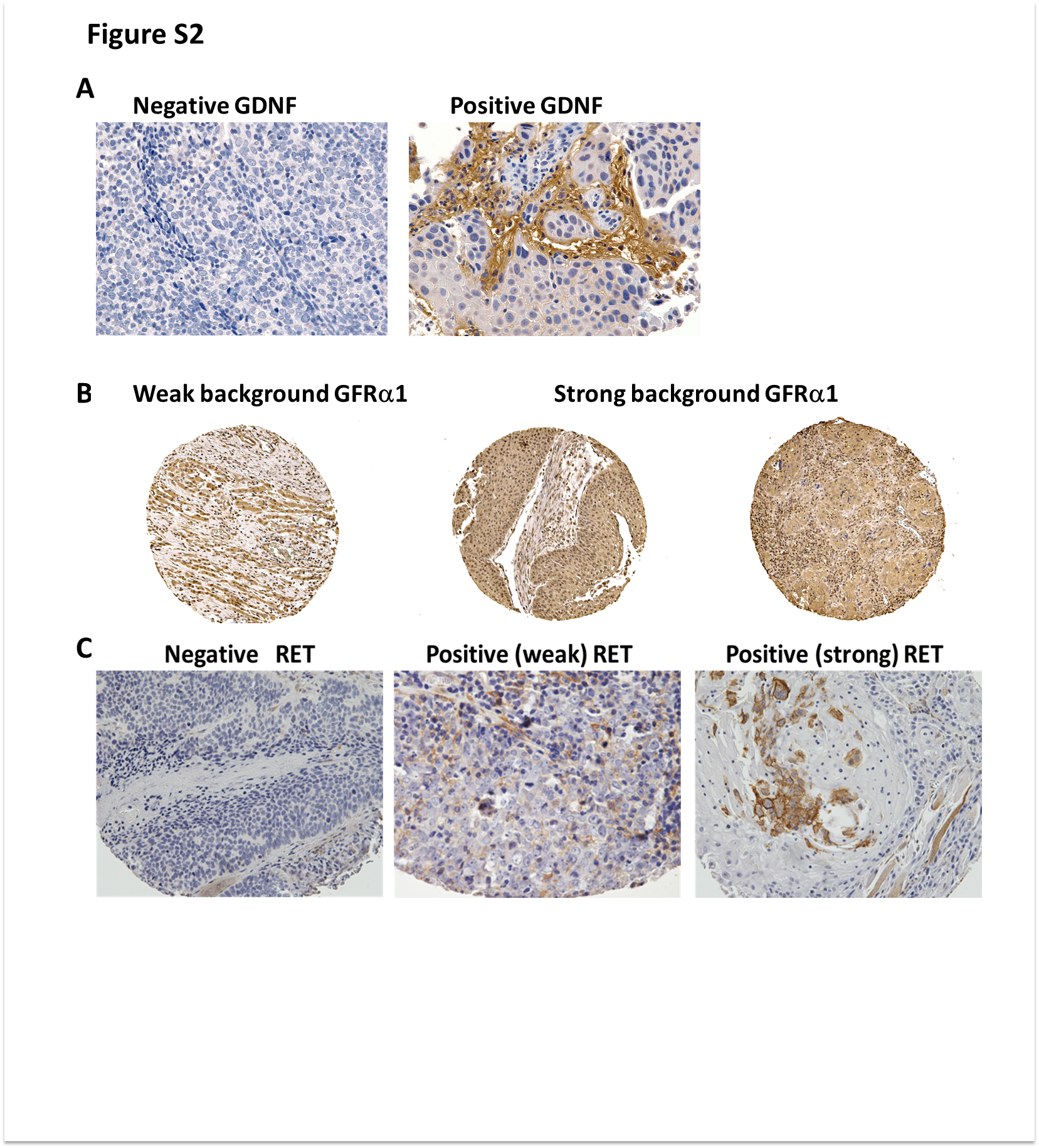
**
