# Supplementary material for: The role of Glial cell derived neurotrophic factor in head and neck cancer
Source: PLoS One. 2020 Feb 21;15(2):e0229311. doi: 10.1371/journal.pone.0229311 (PMC7034888; doi:10.1371/journal.pone.0229311)
Supplement: S3 Fig — (DOCX) [file pone.0229311.s003.docx]

**Supplementary Figure 3.** Kaplan-Meier estimates of overall survival for the SU, WU and TCGA patient cohorts in supplementary table 1.
