# Supplementary material for: The role of Glial cell derived neurotrophic factor in head and neck cancer
Source: PLoS One. 2020 Feb 21;15(2):e0229311. doi: 10.1371/journal.pone.0229311 (PMC7034888; doi:10.1371/journal.pone.0229311)
Supplement: S4 Fig — Overall survival (OS) in HPV-positive tumor in SU cohort (A), WU cohort (C). Overall survival (OS) in HPV-negative tumor in SU cohort (B), WU cohort (D). (DOCX) [file pone.0229311.s004.docx]

**Supplementary Figure 4**. Kaplan-Meier estimates and competing risk analysis of clinical outcomes by HPV and GDNF stromal levels in SU and WU cohorts. Overall survival (OS) in HPV-positive tumor in SU cohort **(A)**, WU cohort **(C)**. Overall survival (OS) in HPV-negative tumor in SU cohort **(B)**, WU cohort **(D)**.

**
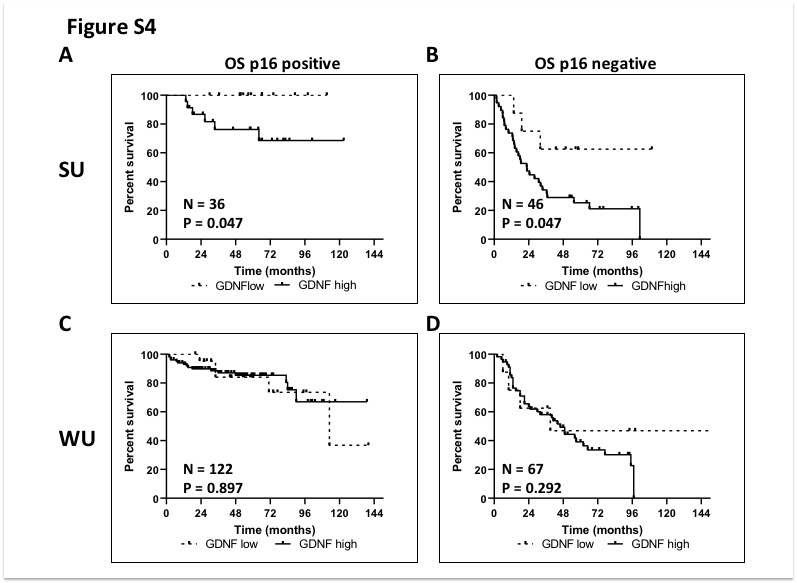
**
