# Supplementary material for: The role of Glial cell derived neurotrophic factor in head and neck cancer
Source: PLoS One. 2020 Feb 21;15(2):e0229311. doi: 10.1371/journal.pone.0229311 (PMC7034888; doi:10.1371/journal.pone.0229311)
Supplement: S1 Table — (DOCX) [file pone.0229311.s005.docx]

**Supplementary Table 1.** Clinical and demographic features of the Stanford University, Washington University and TCGA HNSCC patient cohorts.

**Table S1. Clinical and demographic features of the three cohorts**

| Patient characteristics | Stanford Cohort (%) | Washington University Cohort | TCGA |
| --- | --- | --- | --- |
| Total number of patients | 82 | 189 | 377 |
| Median age [Range] | 60 [20, 94] | 54 [33, 81] | 61 [19, 90] |
| Gender  Male  Female  Missing | 71 (87%)  11 (13%) | 162 (86%)  26 (14%)  1 (0.4%) | 272 (72%)  105 (28%) |
| T-stage  T1-2  T3-4  missing | 36 (44%)  46 (56%) | 104 (55%)  76 (40%)  9 (5%) | 152 (40%)  225 (60%) |
| N-Stage  N0-1  N2  N3  NX | 14 (17%)  54 (66%)  14 (17%) | 62 (33%)  112 (59%)  10 (5%)  5 (3%) | 204 (54%)  134 (35%)  4 (1%)  35 (9%) |
| Stage  I-II  III-IV  Missing | 4 (5%)  78 (95%) | 13 (7%)  171 (90%)  5 (3%) | 97 (26%)  280 (74%) |
| Treatment  Surgery  Surgery + RT (+/- CT)  RT (+/- CT)  missing | 18 (22%)  64 (78%) | 10 (5%)  30 (16%)  145 (77%)  4 (2%) | 75 (20%)  17 (5%)  28 (7%)  253 (67%) |
| P16 status (HPV surrogate)  Positive  Negative  Missing | 36 (44%)  46 (56%) | 122 (65%)  67 (35%) | 13 (3%)  55 (15%)  309 (82%) |
| Median follow-up in months [Range] | 40 [2, 123] | 45 [0, 151] | 23 [1, 211] |
| GDNF stromal staining  Positive  Negative | 61 (74%)  21 (26%) | 159 (84%)  30 (16%) | 185 (49%)  192 (51%) |

The analysis in the study only includes the patients that have no missing values for death, time to OS, T stage, gender, stage, N stage and GDNF.
