# Supplementary material for: The role of Glial cell derived neurotrophic factor in head and neck cancer
Source: PLoS One. 2020 Feb 21;15(2):e0229311. doi: 10.1371/journal.pone.0229311 (PMC7034888; doi:10.1371/journal.pone.0229311)
Supplement: S2 Table — (DOCX) [file pone.0229311.s006.docx]

**Supplementary Table 2.** Statistical analysis of GDNF stromal expression in HPV positive and negative patients in Stanford University cohort.

**Table S2. Statistical analysis of GDNF stromal expression in HPV positive and negative patients in Stanford cohort**

|  | **P16 positive (N=36)** | | | **P16 negative (N=46)** | | |
| --- | --- | --- | --- | --- | --- | --- |
|  | **GDNF negative** | **GDNF positive** | **p-value** | **GDNF negative** | **GDNF positive** | **p-value** |
| **N** | 13 | 23 |  | 8 | 38 |  |
| **OS** | 100% | 87% | 0.0466 | 75% | 47% | 0.0469 |
| **PFS** | 100% | 70% | 0.0191 | 63% | 37% | 0.0465 |
| **DF** | 0% | 9% | 0.2809 | 13% | 18% | 0.4660 |
| **NF** | 0% | 17% | 0.1166 | 0% | 39% | 0.0341 |
| **LF** | 0% | 13% | 0.1114 | 25% | 32% | 0.6332 |

N, patient number; OS, overall survival; PFS, progression-free survival; DF, distal failure; NF, nodal failure; LF, local failure .

For OS and PFS: 2 year survival rate

For DF, NF and LF: 2 year cumulative incidence rate
