# Supplementary material for: The role of Glial cell derived neurotrophic factor in head and neck cancer
Source: PLoS One. 2020 Feb 21;15(2):e0229311. doi: 10.1371/journal.pone.0229311 (PMC7034888; doi:10.1371/journal.pone.0229311)
Supplement: S3 Table — (DOCX) [file pone.0229311.s007.docx]

**Supplementary Table 3.** Statistical analysis of GDNF stromal expression in HPV positive and negative patients in Washington University cohort.

**Table S3. Statistical analysis of GDNF stromal expression in HPV positive and negative patients in Washington University cohort**

|  | **P16 positive (N=122)** | | | **P16 negative (N=67)** | | |
| --- | --- | --- | --- | --- | --- | --- |
|  | **GDNF negative** | **GDNF positive** | **p-value** | **GDNF negative** | **GDNF positive** | **p-value** |
| **N** | 22 | 100 |  | 8 | 59 |  |
| **OS** | 95.2% | 89.8% | 0.8974 | 62.5% | 62.6% | 0.2915 |
| **PFS** | 90.7% | 87.9% | 0.6783 | 71.4% | 55.6% | 0.1344 |
| **DF** | 9.3% | 6.1% | 0.9475 | 0% | 8.9% | 0.2940 |
| **NF** | 0% | 3.0% | 0.8563 | 0% | 12.6% | 0.2249 |
| **LF** | 0% | 2.0% | 0.6562 | 14.3% | 18.0% | 0.7726 |

N, patient number; OS, overall survival; PFS, progression-free survival; DF, distal failure; NF, nodal failure; LF, local failure .

For OS and PFS: 2 year survival rate

For DF, NF and LF: 2 year cumulative incidence rate

There were a total of 157 p16 positive patients but 35 had no GDNF information, which left 122 patients in the analyses.

There were at total of 80 p16 negative patients but 13 had no GDNF information, which left a total of 67 patients in the analyses.
