# Supplementary material for: The role of Glial cell derived neurotrophic factor in head and neck cancer
Source: PLoS One. 2020 Feb 21;15(2):e0229311. doi: 10.1371/journal.pone.0229311 (PMC7034888; doi:10.1371/journal.pone.0229311)
Supplement: S4 Table — (A) For patients with HPV-positive HNSCC in the SU/WU group (N = 150). (B) For patients with HPV-negative HNSCC in the SU/WU group (N = 104). (DOCX) [file pone.0229311.s008.docx]

**Supplementary Table 4.** Multivariate Cox analysis of progression-free survival.

A: For patients with HPV-positive HNSCC in the SU/WU group (N = 150)

| **Parameter** |  | **Reference** | **HR** | **95%CI** | | **p-value** |
| --- | --- | --- | --- | --- | --- | --- |
| **N stage** | N2 | N0/N1 | 1.414 | 0.532 | 3.758 | 0.026 |
| **N stage** | N3 | N0/N1 | 4.462 | 1.333 | 14.936 |  |
| **Treatment** | RT+surgery/ surgery only | RT only | 2.379 | 1.086 | 5.21 | 0.030 |
| **GDNF** | Positive | Negative | 1.802 | 0.737 | 4.405 | 0.197 |

B: For patients with HPV-negative HNSCC in the SU/WU group (N = 104)

| **Parameter** |  | **Reference** | **HR** | **95%CI** | | **p-value** |
| --- | --- | --- | --- | --- | --- | --- |
| **Sex** | Male | Female | 1.037 | 0.532 | 1.037 | 0.915 |
| **Age** |  |  | 1.01 | 0.985 | 1.01 | 0.438 |
| **N stage** | N2 | N0/N1 | 1.214 | 0.723 | 1.214 | 0.246 |
| **N stage** | N3 | N0/N1 | 2.044 | 0.884 | 2.044 |  |
| **T stage** | T3/4 | T1/T2 | 1.832 | 1.041 | 1.832 | 0.036 |
| **Treatment** | RT+surgery/surgery only | RT only | 2.52 | 1.446 | 2.52 | 0.001 |
| **GDNF** | Positive | Negative | 2.778 | 1.168 | 2.778 | **0.021** |
| **SU** | Wash U | Stanford | 1.159 | 0.68 | 1.159 | 0.588 |
