# Supplementary material for: The role of Glial cell derived neurotrophic factor in head and neck cancer
Source: PLoS One. 2020 Feb 21;15(2):e0229311. doi: 10.1371/journal.pone.0229311 (PMC7034888; doi:10.1371/journal.pone.0229311)
Supplement: S5 Table — (DOCX) [file pone.0229311.s009.docx]

**Supplementary Table 5.** Statistical analysis of RET stromal expression in HPV positive and negative patients in Stanford University cohort.

**Table S5. Statistical analysis of RET stromal expression in in HPV positive and negative patients in Stanford cohort**

|  | **P16 positive (N=36)** | | | **P16 negative (N=46)** | | |
| --- | --- | --- | --- | --- | --- | --- |
|  | **Ret negative** | **Ret positive** | **p-value** | **Ret negative** | **Ret positive** | **p-value** |
| **N** | 17 | 19 |  | 30 | 16 |  |
| **OS** | 88% | 95% | 0.3258 | 50% | 56% | 0.1615 |
| **PFS** | 76% | 84% | 0.3744 | 37% | 50% | 0.1349 |
| **DF** | 6% | 5% | 0.9203 | 20% | 13% | 0.3157 |
| **NF** | 12% | 11% | 0.9309 | 40% | 19% | 0.2958 |
| **LF** | 12% | 5% | 0.9041 | 33% | 25% | 0.5736 |

N, patient number; OS, overall survival; PFS, progression-free survival; DF, distal failure; NF, nodal failure; LF, local failure .

For OS and PFS: 2 year survival rate

For DF, NF and LF: 2 year cumulative incidence rate
