# Supplementary material for: The role of Glial cell derived neurotrophic factor in head and neck cancer
Source: PLoS One. 2020 Feb 21;15(2):e0229311. doi: 10.1371/journal.pone.0229311 (PMC7034888; doi:10.1371/journal.pone.0229311)
Supplement: S6 Table — (A) Comparing NCAM gene expression. (B) Comparing GFRα1 expression, and (C) Comparing RET gene expression. (DOCX) [file pone.0229311.s010.docx]

**Supplementary Table 6.** Multivariate Cox analysis of overall survival for HPV-negative HNSCC patients in TCGA (N = 340)

1. Comparing *NCAM* gene expression

| **Parameter** |  | **Reference** | **HR** | **95%CI** | | **p-value** |
| --- | --- | --- | --- | --- | --- | --- |
| **Sex** | Female | Male | 1.097 | 0.775 | 1.554 | 0.600 |
| **Age** |  |  | 1.026 | 1.01 | 1.042 | 0.001 |
| **T stage** | T3/4 | T1/2 | 1.744 | 0.988 | 3.077 | 0.055 |
| **Stage** | Stage 3/4 | 1 / 2 | 0.789 | 0.406 | 1.532 | 0.484 |
| ***NCAM*** | NCAM ≥ 6.6 | NCAM < 6.6 | 1.143 | 0.835 | 1.564 | 0.404 |
| **N stage** | N2/N3 | NX/N0/N1 | 1.881 | 1.302 | 2.681 | < 0.001 |

1. Comparing *GFR-α1* gene expression

| **Parameter** |  | **Reference** | **HR** | **95%CI** | | **p-value** |
| --- | --- | --- | --- | --- | --- | --- |
| **Sex** | Female | Male | 1.076 | 0.757 | 1.53 | 0.683 |
| **Age** |  |  | 1.026 | 1.01 | 1.042 | 0.001 |
| **T stage** | T3/4 | T1/2 | 1.753 | 0.993 | 3.094 | 0.053 |
| **Stage** | Stage 3/4 | 1 / 2 | 0.787 | 0.404 | 1.531 | 0.480 |
| ***GFR-α1*** | *GFR-a1* ≥ 5.6 | *GFR-a1* < 5.6 | 1.148 | 0.839 | 1.571 | 0.388 |
| **N stage** | N2/N3 | NX/N0/N1 | 1.936 | 1.362 | 2.754 | < 0.001 |

1. Comparing *RET* gene expression

| **Parameter** |  | **Reference** | **HR** | **95%CI** | | **p-value** |
| --- | --- | --- | --- | --- | --- | --- |
| **Sex** | Female | Male | 1.105 | 0.777 | 1.572 | 0.577 |
| **Age** |  |  | 1.025 | 1.01 | 1.042 | 0.002 |
| **T stage** | T3/4 | T1/2 | 1.689 | 0.957 | 2.982 | 0.0707 |
| **Stage** | Stage 3/4 | 1 / 2 | 0.804 | 0.414 | 1.559 | 0.518 |
| ***RET*** | *RET* ≥ 13 | *RET* < 13 | 0.982 | 0.715 | 1.348 | 0.909 |
| **N stage** | N2/N3 | NX/N0/N1 | 1.921 | 1.351 | 2.732 | 0< 0.001 |
